# Green Innovation, Corporate Environmental Ethics, and Competitive Advantages of Chinese Automobile Industry During COVID-19: Corporate Environmental Management as Moderator

**DOI:** 10.3389/fpsyg.2022.832895

**Published:** 2022-07-27

**Authors:** Wenhan Wu, Wenzhuo Wu, Kouhua Wu, Chen Ding, Chenya Fan

**Affiliations:** ^1^Southampton Business School, University of Southampton, Southampton, United Kingdom; ^2^School of Economics and Management, Tsinghua University, Beijing, China; ^3^Department of Administrative, YixingWukouhua Purple Sand Art Museum, Yixing, China; ^4^School of Civil Engineering, Southeast University, Nanjing, China

**Keywords:** green innovation, corporate environmental ethics, corporate environmental management, competitive advantages, COVID-19

## Abstract

**Objective:**

The main purpose of this study is to investigate the impact of green product and process innovation on the competitive advantages of the Chinese automobile industry during coronavirus disease 2019 (COVID-19). This study also examined the mediating role of corporate environmental ethics (CEE) and the moderating role of corporate environmental management in the relationship between the green product and process innovation on the competitive advantages of the Chinese automobile industry during COVID-19.

**Methods:**

This study used a quantitative approach of research with the cross-sectional method for the collection of data. This study also used purposive sampling for the collection of data from the production managers of the automobile industry of China. The structural equation modeling-partial least squares (SEM-PLS) is used to analyze the data.

**Results:**

The results of direct effects indicated that green product innovation has a significant and positive effect on the corporate advantages (β = 0.294, *t* = 2.868) and green process innovation also has a significant and positive effect on the corporate advantages (β = 0.350, *t* = 3.276). Moreover, green product innovation has also a significant effect on corporate advantages (β = 0.334, *t* = 4.258) and green product innovation has also a significant effect on corporate advantages (β = 0.269, *t* = 3.202).

**Significance:**

The research in this domain about the antecedents of green innovation is still minimal in the previous literature. One of the antecedents of the green innovation, corporate environmental ethics is discussed in this study; thus, it provides the understanding of green innovation as the mediator which would mediate the relationship between corporate environmental ethics and competitive advantage in the auto manufacturing industry of China.

**Novelty:**

This study is among very few to examine the relationship between green innovation, corporate environmental ethics, corporate environmental management, and competitive advantages of the Chinese automobile industry during COVID-19.

## Background

Green innovation is considered now as one of the crucial and effective strategies for sustainable development of manufacturing industries due to the increasing stress on environmental issues. The investment in environmental projects was considered as a wastage of resources in the earlier period. But now, new trends for the firms have been set as new strict rules and regulations have been formulated for environmental issues, which have completely rotated the concepts of competitiveness for organizations (Xie et al., 2019). Green products and green processes are two categories of green innovation, encompassing the innovative technologies regarding the reduction of unnecessary energy consumptions, averting pollution, recycling of the wastes, corporate environmental management (CEM), or green product designs (Chang, 2018). If organizations want to practice green innovation vigorously, the advantage can be taken from low cost and differentiation, which may alter the rules for competition (Gürlek and Tuna, 2018; Xie et al., 2019). Hence, green innovation is adopted more nowadays, as it is considered more profitable for an organizations in the present era (Song and Yu, 2018; Xie et al., 2019). Focusing on how to be green innovation can lead to exploitation of new market opportunities, consistent innovativenesss, and creation of more wealth (Khan, 2019). The designs and packaging of products of the company can indulge the green concept with the idea of protecting the environment, which can increase their repute of differentiation (Chang, 2018; Wong et al., 2020). While practicing environmental management, not only the company would be able to avoid persecution and complaints but might become able to boost the efficiency of production, establish new environmental markets and, thus, it can contribute to greater green innovation capabilities (Rehman et al., 2021).

According to the definition of Danso et al. (2019) and Galbreath et al. (2020), competitive advantages are a particular situation in which no other market competitors can imitate the executed competitive strategies of a firm. It has been discussed in the prior research that regarding the Chinese electronic industry, there is a positive relationship between green innovation and competitiveness (Chang, 2018). There is a greater impact of green innovation on product value which can balance the cost of investment in environmental causes. Green innovation ultimately leads to superior productivity and then the organization excels in its goal attainment (Xie et al., 2019). As the global environment is changing drastically, environmental management is getting crucial for the companies, and green innovation is consistently taking maximum attention of the firms for its implication. Hence, green innovation is a bright source for the economic and environmental development of a firm. China has implemented environmental regulations which can be satisfied by green innovation and, hence, can improve the environmental management performance of an organization (Chang, 2018). Though the research in this domain about the antecedents of green innovation is still minimal in the previous literature. One of the antecedents of green innovation, corporate environmental ethics (CEE) is discussed in this study, thus, it provides the understanding of green innovation as the mediator which would mediate the relationship between CEE and competitive advantage in the auto manufacturing industry of China.

Chinese automobile during the last two decades has emerged as one of the world’s leading automobile industries. According to the annual production report 2009 of the Chinese automobile industry in terms of the total number of units produced, the Chinese automobile industry has outnumbered the combined production of the European Union or that of the United States and Japan combined. China became the largest automobile market in the world. China is the biggest market to the automobile industry as around 80% of the auto supply chain is connected to China. During coronavirus disease 2019 (COVID-19), the Chinese auto sale has declined by 18%. According to the Chinese passenger association, the sale could further decline by 40%.

Coronavirus disease 2019 spreading the globe, vandalization lives, business, and economies. Consequently, placing an enormous pressure on the public health as well as on corporations and organizations for being socially, and environmentally reasonable. Today, CEE and CEM are the new tools for hoteliers when they encounter an irresistible force of urgent nature, which demands an urgent and timely investigation to explore the external factors. Mentioning the argument of Adner et al. (2019), they state that a culture of a firm can provide a sustainable competitive advantage if that culture is rare, imperfectly imitable, and valuable. Retention of legitimacy is not the only sole purpose of green management, but it also acts as a center hub of an organization, which plays key role in achieving sustainable competitive advantage (Hervas, 2019). Organizational culture holds CEE as a key factor leading to innovative practices (Kassar and Singh, 2019). The expectation for ethical behavior and corporate value is formalized by the CEE and so it proves to be an incentive for competitive advantage and green innovation. Following is the structure of this study. Second section comprises five hypotheses and literature review. The third section comprises the methodology, data collection, and sample selection and constructs measurement. The fourth segment encompasses reliability of the measurement, factor analysis, descriptive statistics, correlation coefficients between constructs, and the results are demonstrated for the measurement and structural model. This study exhibits discussions about the findings and implications and highlights the possible directions for conducting future studies in the fifth segment.

## Literature Review

### Green Innovation

In today’s competitive world, businesses have been using innovation as a key tool for increasing market share and their survival in the market. Innovation facilitates firms in attracting potential customers, achieving a competitive advantage, and improving the firm’s position in the market (Richter and Hauff, 2022). Due to the significance of innovation, several tourism researchers are taking a significant interest in this area (Novelli et al., 2006). The majority of the innovation-based studies have been conducted using the Schumpeterian innovation theory. This theory defines innovation as the transformation of existing knowledge or creating new knowledge within the organization (Schumpeter and Nichol, 1934). In Schumpeter (1942) study, he examined innovation using the economic development perspective. In his study, he presented a new concept, i.e., creative destruction and defined it as the continuous process of development, i.e., breaking down the old structures and renewing the economy by developing new structures. The creative destruction approach posits that organization which fails to emphasize and integrate innovation is likely to face organizational inertia, while the ones who integrate innovation becomes the driving force to reach economic development. According to the Schumpeterian perspective, there are five types of innovation, namely, new processes, new products, new industrial structures, new input sources, and new markets (Schumpeter and Nichol, 1934). Many prior studies (Camisón and Monfort-Mir, 2012; Rodríguez-Caballero et al., 2014; Rodriguez-Sanchez et al., 2019) have also adopted this perspective. Such innovation types can be defined as: the process innovation is defined as the backstage innovations aiming to improve the efficiency and productivity of the firm (Hjalager, 2010). Product innovation is defined as the changes that the customers observe directly and then accept the changes as new (Gomezelj, 2016). Organizational innovation is the process of developing new business models and new management techniques. On the other hand, the term marketing innovation is the integration of new marketing strategies and techniques to the business processes (Camisón and Monfort-Mir, 2012).

### Corporate Environmental Management

External environmental pressures may lead firms toward implementing environmental management. According to the Neoclassical economists, the main goal of companies is to maximize the wealth of the shareholders (Friedman, 1970; Tampakoudis et al., 2021), while the institutional theory is concerned more about the external institutions and how they influence firms’ strategies (Hillis et al., 2018). According to this theory, profit maximization is not the only objective of the companies. Companies integrate green products and innovation to earn external institutions’ trust. The resource-based view (RBV) states that the companies’ key capabilities and resources result in competitive advantage (Zahra, 2021). According to the RBV, environmental social responsibility is one of the key capabilities of the firms, which brings about sustained competitive advantage to the firm (Hart, 1995). Stakeholder activism, international environmental regulation, competitive pressures, environmentalism, activism, and national environmental policies are the environmental policies that affect the firms’ operations (Chen et al., 2020). Therefore, to get aligned with consumer environmentalism and international environmental regulations, companies need to integrate environmental management into their operations. Thus, when it comes to a firm’s strategies, environmental management is considered as one of the unique firm capabilities and a key factor in developing strategies (Suryanto et al., 2018). The corporate social responsibility literature posits that the social responsibilities of the companies emphasize working toward the companies’ economic objectives (Hillis et al., 2018). Besides, integrating corporate environmental management also helps in shaping the firm’s environmental rules and enables them to gain the first-mover advantage (Tampakoudis et al., 2021). The implementation of environmental management also involves the strict implementation of environmental standards into green processes and products thereby implementing a high barrier to entry. Obtaining support from key stakeholders and external institutions facilitate firms in achieving competitive advantage. Thus, prior literature concerning stakeholder theory, institutional theory, corporate social responsibility, and the RBV provides support on the positive association between corporate environmental activities and competitive advantage (Dmytriyev et al., 2021).

### Competitive Advantage

Many small and medium enterprises (SMEs) share a common goal, i.e., aiming to achieve a strong competitive advantage. SMEs tend to pay more attention to competitive advantage, as it leads the firm toward higher performance (Kanten et al., 2015; Yeşiltaş et al., 2020). According to the RBV, the key drivers to business performance and competitive advantages are the unique capability and resources of the firm. Leonidou et al. (2013) suggest green organizational culture and green innovation as the potential drivers to achieve competitive advantage. Comparative positional superiority is the key factor that needs to be emphasized for achieving competitive advantage, as it enables to attain better performance as compared to the market competitors. Superior position can be attained by firms in comparison to their competitors simply by achieving low operating costs. Contrarily, adopting innovative production and product processes also helps firms to excel from the competitors in the market (Zhou et al., 2009). This study described competitive advantage using the RBV just as suggested by the earlier scholars (Cheraghalizadeh and Tümer, 2017) and defined competitive advantage as the position of an organization where the competitors cannot imitate those actions and processes, which provide sustainable benefits to the firm. This definition is appropriate because the comparative positional superiority serves as the indicator of the firm’s competitive advantage.

### Corporate Environmental Ethics

Corporate environmental ethics refers to the firm’s fundamental ethical attitude, belief, and mindset about the environmental aspect (Han et al., 2019). A firm’s expectations and values concerning the environment are shaped through CEE (Zou et al., 2019). There are six aspects of CEE, namely, ethics committees, ethics codes, ethics officers, disciplinary processes, ethics training programs, and ethics communication systems (Remišová et al., 2019). The natural RBV posits that firms develop green capability and culture due to the challenges and constraints presented by their natural environment. The green capabilities and green culture are difficult to be imitated and provide benefits to the firm at three different levels, i.e., sustainable development, product stewardship, and pollution prevention. The way environmental problems are dealt with, thus, explains the overall competitiveness of a firm or an industry (Ma et al., 2017). This implies that firm performance can be greatly improved through CEE. CEE may result in cost reduction for the firms. Menguc et al. (2010) argue that firms which follow the authoritarian regulation and pollution control standards are less likely to face punishment or penalty for disobeying the environmental standards. There is susceptibility among the firms to integrate environmental consideration in terms of process and product design. Thus, firms tend to adopt efficient ways of product and process design, i.e., utilizing energy, labor, and raw materials to minimize the cost of adopting environment-friendly behavior.

### Hypothesis Development

Competitive advantages are referred to those strategies executed by an organization that is not imitable by any of the competitors of the firm, even the competitors are not capable of achieving those advantages which are achieved by the competitive strategy executing company (Danso et al., 2019; Qershi, 2019; Galbreath et al., 2020). The unique resources which are exploited by a firm to gain innovation and competitive advantage include the following characteristics: rare, imperfectly imitable, valuable, and unsubstitutable (Nanath and Pillai, 2017; Gürlek and Tuna, 2018). Due to the innovative approach, isolation mechanisms are generated through which advantages can be grasped and profit margins are being protected. In this present era of the knowledge economy, innovation is a key to competitive advantage (Chau, 2017). Companies enable themselves to gain long-term benefits by structuring and utilizing their capabilities through innovation. With the help of successful innovation, it becomes difficult for the competitors to replicate the strategies, which result in sustainable competitive advantage. Those organizations which are excelling in green innovation are capable of obtaining competitive advantage and green innovation they can maintain their repute by selling environmental products and even contributing to new market creation (Chang, 2018; Xie et al., 2019; Wong et al., 2020). The organizations which are willing to invest in environmental causes and green innovation are not only capable of waste reduction, but are also capable of boosting their productivity, improving their repute and image; therefore, growing the competitive advantage of a company relating to the environmental trends of international rules of protection of the environment and relevant to customer’s view (Chang, 2018; Xie et al., 2019; Agustia, 2020). An organization can boost up its green image by adopting green product innovation (GPrdI). Thus, GPrdI can lead toward the achievement of competitive advantage (Chang, 2018). Not only in terms of competitive advantage, but also it is helpful in the reduction of cost. It has been mentioned in prior literature that pollution is the proof of incompetent use of resources (Xie et al., 2019). Along with the prevention of waste, green process innovation (GPrsI) is also responsible for the improvement in the efficient use of resources (Xie et al., 2019; Galbreath et al., 2020).

Productivity of the resources is boosted from GPrsI through energy decreasing, material saving, reduction in resources, and waste recycling (Rehman et al., 2021). The benefit of GPrsI is not only about the prevention of the expense of pollution, rather it also concerns the reduction of expenses generated through resources and cuts off the inclusive cost (Nanath and Pillai, 2017). GPrsI can be carried on by the organizations to boost up their productivity and efficiency in the manufacturing process, which may enable the organization to get the benefit of low cost (Rehman et al., 2021). Along with all other benefits, it is easier to satisfy stakeholders through GPrsI (Rehman et al., 2021). Hence, competitive advantage can be gained through GPrsI adopted by the organizations (Chang, 2018). Contrarily, the adoption of green innovation also leads to cost reduction by recycling waste, less utilization of resources, ensuring less energy consumption, and material saving (Hart, 1995; Eiadat et al., 2008). Hence, by lowering the cost, green innovation provides firms to achieve a competitive advantage (Chang, 2011). There are limited studies available in context to green innovation in the hotel industry and the findings of these studies are presented as follows. In Jacob et al.’s (2010) study, they found that green innovation-related tourism firms in the Balearic Islands enjoy high competitiveness. Until now, only a few studies have addressed the causal association between competitive advantage and green innovation in the hotel industry. However, a few other related topics have also been studied by the researchers, such as a positive impact of environmental management was found on the competitive advantage in the hotel industry in Spain (Molina-Azorín et al., 2015). Similarly, Leonidou et al. (2013) reported that environmental management-related capabilities and resources positively influence the competitive advantage. Furthermore, in Fraj et al.’s (2015) study, they found a positive impact of environmental strategy on organizational competitiveness. The present study is in line with the Leonidou et al.’s (2013) study, both emphasize the firm’s RBV and suggest that green innovation capability is the firm’s unique capability that leads the firm toward competitive advantage. Thus, we propose the following hypothesis based on the above arguments and discussion:

***Hypothesis 1 (H1)** GPrdI has a positive impact on competitive advantages.*
***Hypothesis 2 (H2)** GPrsI has a positive impact on competitive advantages.*

Corporate environmental ethics is the complete belief of ethical practices, values, and environment concerning rules within an organization. There are six elements of CEE: ethics committees, ethics officers, ethics codes, ethics communication systems, disciplinary processes, and ethics training programs (Han et al., 2019). Firms must be paying attention to their objective of sustainable development by taking the global effects of environmental issues into consideration. Values and expectations of a firm can be validated through CEE. Along with the prevention of the threat of protests, those companies which have a high benchmark of environmental ethics can make their repute better than those which have a low standard of environmental ethics (Chang, 2018). Thus, long-lasting advantages can be gained from effective environmental management. Firms are motivated to get a niche place in a market so that their rivals may not replicate their strategies and the firms enjoy their competitive edge, this situation is referred to as a competitive advantage (Xie et al., 2019; Galbreath et al., 2020). Not only the rules and regulations regarding the environment are met, but the companies can make their competitors lag due to CEE. When companies are devoted to maintaining their intangible assets, they become capable of sustaining their competitive advantage (Rehman et al., 2021). Intangible assets of a firm might be comprised of CEE. It can be observed that those companies which practice environmental management can set themselves up to such a standard where no rivals can match them and they can easily sustain their competitive advantage by deploying particular competitive strategies. Hence, the following hypothesis can be proposed by the present study on the prior mentioned arguments:

***Hypothesis 3 (H3)** CEE has a positive impact on competitive advantages.*
***Hypothesis 4 (H4)** CEE mediates the relationship between the greed product innovation and competitive advantages.*
***Hypothesis 5 (H5)** CEE mediates the relationship between the greed product innovation and competitive advantages.*

The external pressures of the environment may be a vital cause to adopt CEM. Nevertheless, it has been stated by neoclassical economics that the maximization of wealth should be the prime objective of an organization (Maqbool and Zameer, 2018), But the institutional theory states that a firm should also pay attention to the external forces, which hold an adequate impact on the strategies of that organization (Sarkis and Zhu, 2018). This further directs the firms to the point that firm’s ultimate social goal is not only the maximization of profit, but also to fulfill the requirements of legitimacy as it asserts certain pressures. Companies can use green innovation and put their products under the umbrella of green factors so that they can become capable of meeting the requirements of external pressures regarding legitimacy. Referring to the RBV, it has been explicated that the main resources and competencies can lead to competitive advantage (Nanath and Pillai, 2017; Danso et al., 2019). It has been suggested by the RBV that a firm can sustain its competitive advantage by making its corporate social responsibility a key competency (Wong et al., 2020). The operations of a company can be impacted by several forces which might include competitive pressures, stakeholder activism and environmentalism, and policies and regulations of national level and international level. Therefore, the firms should work on environmental management to cope with the international standards and policies regarding the environment. Thus, a set of firm’s strategies might include environmental management as a key element, additionally, from an RBV perspective, it should be considered as a crucial competency for a firm (Wong et al., 2020). The previous studies conducted on corporate social responsibility indicate that the economic goals of a firm can be achieved by fulfilling its social responsibilities. The organizations are intended to be socially responsible and because of this, they in. Though it is noticed that environmental management does not serve short-term objectives but it complies to improve economic goals in the long-run objectives of a company. Moreover, it has been advised to the organizations that they should be considerate of their key stakeholders while formulating policies and strategies so that they could capture their trust and assistance (Mhlanga and Moloi, 2020). It would be tough for the organizations to merge environmental management with their strategies if the organizations continue to give prime importance only to the economic goals. The companies must be focusing on non-profit objectives and should be concerned about external institutions and stakeholders (Todaro et al., 2020). Companies become capable of formulating competitive policies and ultimately firms are allowed to get first-mover benefits. Firms are forced by the environmental management standards that the green factor should be strictly incorporated in the products and services and thus generating high barriers to entry. To obtain a competitive advantage, firms can take assistance from their key stakeholders and external institutions. Therefore, referring to corporate social responsibility, institutional theory, RBV, and stakeholder theory, the arguments can support the positive relationship between competitive advantage and corporate environmental practices (Todaro et al., 2020).

***Hypothesis 6 (H6)** CEM moderates the relationship between the greed product innovation and competitive advantages.*
***Hypothesis 7 (H7)** CEM moderates the relationship between the greed product innovation and competitive advantages*.

## Methodology

As this study concerns competitive advantages and environmental ethics in the automobile industry in China, the sample contains employees of the automobile industry. The targeted respondents were the production managers of the automobile industry. In the selection of sample size, this study used the inferential statistics of Kyriazos (2018). According to Kyriazos (2018), a sample of fewer than fifty respondents is considered weaker, a sample of one hundred is reflected a weak sample size, two hundred respondents assumed an adequate sample size, and a sample of three hundred respondents is considered a good sample size. This study also employed the Gpower software in the calculation of minimum required sample size. The model of this study consists of 2 predictors for the independent construct, i.e., competitive advantages. The Gpower software confirmed that the three hundreds sample size is required. Therefore, this study set the sample size of three hundreds respondents to collect the data. This study used quantitative approach of research with cross-sectional method for the collection of data. This study also used the purposive sampling for the collection of data from the production managers of automobile industry of China. The participation of the production managers in this study was based on volunteer basis. Structured questionnaire was used to gather the data from the respondents.

### Measurement

The scale item for all the constructs were adapted from the previous studies and measured on a 5-point Likert scale. The scale of CEE, competitive advantages, and green innovation are adopted from the study of Chang (2011). Additionally, the scale of corporate environmental management is adopted from the study of Latan et al. (2018).

## Analysis and Results

**FIGURE 1:**
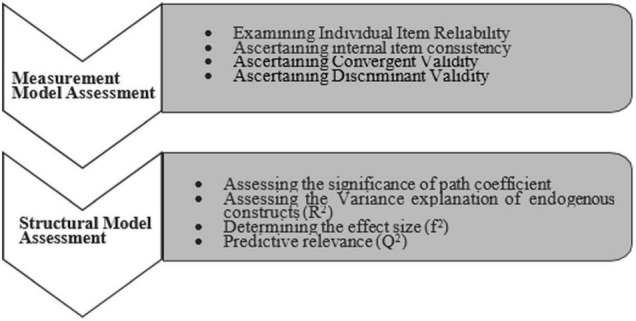
A two-step process of partial least squares (PLS) path model assessment (Henseler et al., 2009).

“Partial least squares” (PLS) method of analysis was employed for the analysis of research model using the smart-PLS (Ringle et al., 2015; Shehzadi et al., 2020; Raoof et al., 2021). This study followed the recommended two-stage analytical technique (Ramayah et al., 2017; Hair et al., 2019). This study examined the measurement model followed by the testing of the structural model (Shiau et al., 2019). Figure 1 elucidates the two-step PLS-structural equation modeling (SEM) process.

### Measurement Model Evaluation

**FIGURE 2:**
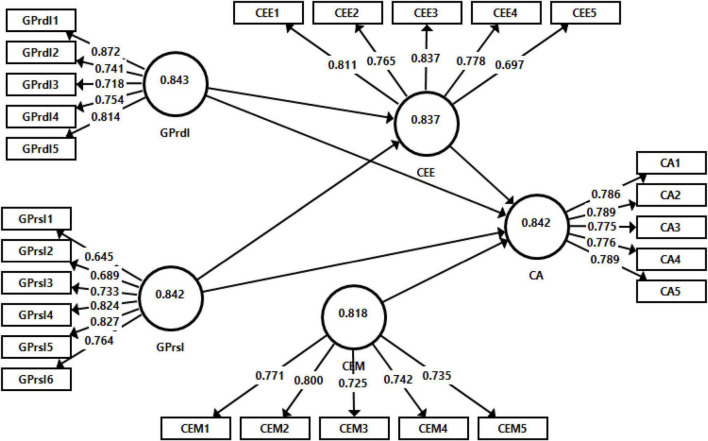
Measurement model assessment.

Two forms of validity were estimated by the examination of the measurement model, i.e., convergent and discriminant validity. In a measurement model, convergent validity is generally discovered by investigating the outer loadings, average variance extracted (AVE), and the composite reliability (CR) (Shiau et al., 2019; Abdulmuhsin et al., 2021). To establish the convergent validity, value of loadings should be higher than 0.5 and the CR and AVE values should be greater than 0.7 and 0.5, respectively (Ringle et al., 2015; Nawaz et al., 2019; Basheer et al., 2021). The discriminant validity is examined by the heterotrait-monotrait (HTMT) ratio and the method of Henseler (2017). The output of measurement model is given in Figure 2 and Tables 1–3.

**TABLE 1:** Internal consistency, convergent validity, composite reliability, and average variance extracted (AVE).

Construct	Indicators	Loadings	Cronbach’s alpha	Composite reliability	AVE
Competitive advantages	CA1	0.786	0.842	0.888	0.613
	CA2	0.789			
	CA3	0.775			
	CA4	0.776			
	CA5	0.789			
CEE	CEE1	0.811	0.837	0.885	0.607
	CEE2	0.765			
	CEE3	0.837			
	CEE4	0.778			
	CEE5	0.697			
CEM	CEM1	0.771	0.818	0.869	0.570
	CEM2	0.800			
	CEM3	0.725			
	CEM4	0.742			
	CEM5	0.735			
GPrdI	GPrdI1	0.872	0.843	0.887	0.611
	GPrdI2	0.741			
	GPrdI3	0.718			
	GPrdI4	0.754			
	GPrdI5	0.814			
GPrsI	GPrsI1	0.645	0.842	0.884	0.562
	GPrsI2	0.689			
	GPrsI3	0.733			
	GPrsI4	0.824			
	GPrsI5	0.827			
	GPrsI6	0.764			

**TABLE 2:** Fornell–Larcker criterion.

	CA	CEE	CEM	GPrdI	GPrsI
CA	0.783				
CEE	0.604	0.779			
CEM	0.740	0.726	0.755		
GPrdI	0.685	0.541	0.619	0.782	
GPrsI	0.642	0.557	0.670	0.705	0.750

**TABLE 3:** Heterotrait-monotrait (HTMT) ratio.

	CA	CEE	CEM	GPrdI	GPrsI
CA					
CEE	0.715				
CEM	0.753	0.793			
GPrdI	0.783	0.620	0.717		
GPrsI	0.763	0.655	0.821	0.825	

Table 1 elucidates that the loadings of all the items are higher than 0.6, the value of CR for all the variables are above 0.7, and the value of AVE are also above 0.5 as recommended by Ramayah et al. (2017) and Hair et al. (2019). Hence, this study establishes the convergent validity.

According to Henseler (2017) discriminant validity is tested by the matching of correlations among the variables and AVE square root of that variable. Referring to Table 2, the square root of the AVEs is higher than the correlations of constructs.

Heterotrait-monotrait ratio also shows that this study established the discriminant validity because all the values of HTMT ratio are lower than 0.85 (referring Table 3). Overall, both the convergent and discriminant validities of the measures in this study are established.

### Structural Model Evaluation

**FIGURE 3:**
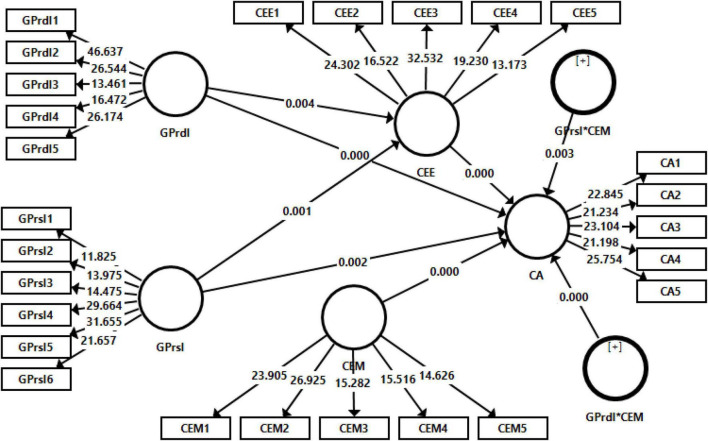
Structural model assessment.

A bootstrapping technique was employed to check the significance of the path coefficients (Shiau et al., 2019). To examine the *t*-values, a bootstrapping method with 1,000 resamples was used. The output of structural model is given in Figure 3 and Tables 4–6.

**TABLE 4:** Structural model assessment (direct effect results and decision).

Hypotheses	Relationship	Beta	STD	*T*-value	*P*-values
H_1_	GPrdI - > CEE	0.294	0.102	2.868	0.004
H_2_	GPrsI - > CEE	0.350	0.107	3.276	0.001
H_3_	GPrdI - > CA	0.334	0.078	4.258	0.000
H_4_	GPrsI - > CA	0.269	0.084	3.202	0.002

**TABLE 5:** Structural model assessment indirect effect (mediation effects).

Hypotheses	Relationship	Beta	STD	*T*-value	*P*-values
H_5_	GPrdI- > CEE - > CA	0.350	0.107	3.276	0.000
H_6_	GPrsI- > CEE - > CA	0.298	0.101	2.950	0.007

**TABLE 6:** Structural model assessment (moderation effects).

Hypotheses	Relationship	Beta	STD	*T*-value	*P*-values
H_7_	GPrdI*CEM - > CA	0.342	0.082	4.348	0.000
H_8_	GPrsI*CEM - > CA	0.313	0.069	4.568	0.003

Table 4 shows the results of direct effects. Results indicated that GPrdI has significantly and positively effect to the corporate advantages (β = 0.294, *t* = 2.868) and GPrsI also has significant and positive effect to the corporate advantages (β = 0.350, *t* = 3.276). Moreover, GPrdI has also a significant effect on corporate advantages (β = 0.334, *t* = 4.258) and GPrdI has also a significant effect on corporate advantages (β = 0.269, *t* = 3.202). Hence, H1, H2, H3, and H4 are supported.

Table 5 indicates the results of mediating role of CEE between the relationship of GPrdI and GPrsI with corporate advantages. Results shows that CEE significantly mediates the relationship of GPrdI with corporate advantages (β = 0.350, *t* = 3.276). It also shows that CEE has significant mediation role between the relationship of GPrsI with corporate advantages (β = 0.298, *t* = 2.950).

Table 6 shows the results of moderating role of CEM on the relationship of GPrdI and GPrsI with corporate advantages. Findings indicated that CEM significantly moderates the relationship of GPrdI with corporate advantages (β = 0.342, *t* = 4.348). Moreover, results also indicated that CEM has significant moderation role on the relationship of GPrsI and corporate advantages (β = 0.313, *t* = 4.568).

## Discussion

The results are in line with the propositions of the RBV theory; according to the RBV, businesses achieve a competitive advantage due to their resources and internal capabilities. Besides, the results of this study also suggest that internal capabilities also bring about sustainable development, pollution control, and provide firms with the solution to several environmental issues. It implies green innovation as the capability, which allows the automobile companies to gain a competitive advantage. In addition, green innovation also provides firms with lower cost and differentiation advantages. The results also highlight that during COVID-19, by improving the quality and design of the product, automobile companies can create differentiated products and services through green innovation, thereby making them unique from their competitors in the market. Consequently, products are sold with a higher profit margin by charging higher prices (Liao et al., 2016). Furthermore, green innovation helps to create isolation mechanisms that are concerned with greater profitability and achievements of the firm. This study categorizes green innovation in two main divisions: GPrsI and GPrdI. The two sources of obtaining competitive advantages have been mentioned as low cost and differentiation (Galbreath et al., 2020). Differentiation strategies are helpful in establishment of unique products by giving them completely unique characteristics. Companies might become capable of setting off their environmental investments with differentiation costs. Product design, reliability, and quality can be improved by deploying GPrdI and due the concerns for environmental issues, the corporations can generate high profit margins and offer high price for green products.

In addition, the findings of this research indicate that during COVID-19, green innovation contributes to the development of isolation mechanisms that are concerned with the firm’s increased profitability and successes. This research classifies GPrsI and GPdI as the two primary categories of green innovation. Low cost and differentiation are the two sources of competitive advantage that have been identified (Galbreath et al., 2020). Differentiation techniques facilitate the development of distinctive goods by endowing them with distinctive qualities. Companies may eventually be allowed to deduct their environmental efforts using differentiation charges. By using GPrdI, product design, dependability, and quality may be enhanced, and owing to environmental concerns, firms can achieve large profit margins and charge a premium for green goods.

Environmental ethics led resource productivity results in increased productivity for the firm. The current study found natural environment as the key criterion for evaluating official promotion in China and firms which consider green aspect are likely to receive the required financial resources from the government because of their good relationship with the government. With regard to resource allocation, Chinese government imposes interventions in the form of natural resource distribution, price setting for the scare resources and assets, distributing bank loan, and exemption and tax reductions. Furthermore, CEE also enable to obtain lower cost capital, tax preference and bank loan, and natural resources. Hence, environment ethics present cost advantage firms with the benefit of enjoying cost reduction in obtaining financial capital and natural resources and cutting the punishment expenditure.

With an intent to become socially responsible, companies tend to adopt environmental management. In the short run, environmental management does not ensure profit gains; however, in the long run it may result in economic payoffs. Furthermore, according to the stakeholder theory, firms are required to consider their stakeholders’ interests while developing strategies as it helps in gaining support and trust from the stakeholders. The results argue that solely emphasizing the economic goals makes it impossible for the firm to adopt environmental management. Therefore, a sustainable long-term approach must be adopted by the firms concerning the stakeholders’ and institutional pressures and non-economic goals.

This study has broadly studied innovation, but still less attention has been paid to green innovation. Thus, the aim of this study is to study the concept of innovation in context to the green perspective. With regard to the Schumpeterian view, process and product innovation can successfully be implemented to the green innovation. In the manufacturing industry, green innovation can be broken down into process innovation and product innovation; however, in the case of the Chinese automobile industry, it is somehow difficult for separating the consumption and production of the Chinese auto industry. Hence, the results of this study explain that during COVID-19 green innovation as integrating innovation into the product and processes to minimize ecological footprints and achieve environmental objectives (Lin et al., 2014). In this study, the concept of green innovation is described as the innovation in the production processes and the product, where production process innovation may involve the ways that can be used for pollution prevention, green design, and energy saving.

## Conclusion

This study deals with the topic on issues of green innovation, competitive advantages, and CEE, together, which makes them emerge into a new subject of “green management” considered about the issue of economic development as well as the protection of the environment. Many manufacturing firms in China have lack of resources and, hence, they are not up to mark for maintaining their standards according to the environmental protection policies. As the organizations in China cannot hold any compliance with the international environmental regulations, it would harm them in every possible way. Nonetheless, the present study found out that if a firm is consistent about investing more in CEE, Chinese manufacturing industry would witness improvement in their GPrdI and competitive advantage (Farza et al., 2021). Thus, these findings can be utilized by the manufacturing industries in China. By observing the environmentalism approach of consumers and strict rules of international level, it is advisable for the firms not to avoid their environmental duties. The external environmental pressures and increasing trends can become a driving force for the firms to engage themselves in environmental ethics leading to green innovation and ultimately moving forward to achieve competitive advantage.

### Future Recommendations

This study has made China as its center of research, the future researchers can make their studies more contextual by focusing on other areas as well. To test the similar hypotheses in other countries according to their context would be a captivating issue. The future studies may make other countries as their context of research which may be helpful in generalization of the findings around the globe. This study is only demonstrating the cross-sectional data through employing survey methods comprising of questionnaires, so no vigorous changes of green innovation, environmental ethics, and competitive advantages have been shown in different developmental stages of industry in China through longitudinal data. Thus, variations in environmental ethics, green innovation, and competitive advantages can be observed through longitudinal study by future researchers in various developmental stages of auto manufacturing industry of China. Finally, this study intends to fulfill the purpose of contributing to managers of auto manufacturing industry of China, their researchers and policy formulators which may further contribute to their respective areas.

## Data Availability Statement

The original contributions presented in the study are included in the article/supplementary material, further inquiries can be directed to the corresponding author/s.

## Ethics Statement

Ethical review and approval was not required for the study on human participants in accordance with the local legislation and institutional requirements. Written informed consent for participation was not required for this study in accordance with the national legislation and the institutional requirements.

## Author Contributions

All authors listed have made a substantial, direct, and intellectual contribution to the work, and approved it for publication.
